# The Association between Nutritional Status and Length of Hospital Stay among Patients with Hypertension

**DOI:** 10.3390/ijerph19105827

**Published:** 2022-05-10

**Authors:** Michał Czapla, Raúl Juárez-Vela, Katarzyna Łokieć, Marta Wleklik, Piotr Karniej, Jacek Smereka

**Affiliations:** 1 Laboratory for Experimental Medicine and Innovative Technologies, Department of Emergency Medical Service, Wroclaw Medical University, 51-616 Wroclaw, Poland; michal.czapla@umw.edu.pl (M.C.); jacek.smereka@umw.edu.pl (J.S.); 2 Institute of Heart Diseases, University Hospital, 50-566 Wroclaw, Poland; 3 Group of Research in Care (GRUPAC), Faculty of Nursing, University of La Rioja, 26006 Logroño, Spain; piotr@karniej.pl; 4 Department of Propaedeutic of Civilization Diseases, Medical University of Lodz, 90-251 Lodz, Poland; katarzyna.lokiec@umed.lodz.pl; 5 Department of Nursing and Obstetrics, Faculty of Health Sciences, Wroclaw Medical University, 51-618 Wroclaw, Poland; marta.wleklik@umw.edu.pl; 6 Faculty of Finance and Management, WSB University in Wrocław, 53-609 Wroclaw, Poland

**Keywords:** hypertension, malnutrition, nutritional status, obesity, BMI, length of hospital stay

## Abstract

Background: Nutritional status is related to the prognosis and length of hospital stay (LOS) of patients with hypertension (HT). This study aimed to assess how nutritional status and body mass index (BMI) affect LOS for patients with hypertension. Method: We performed a retrospective analysis of 586 medical records of patients who had been admitted to the Institute of Heart Diseases of the University Clinical Hospital in Wroclaw, Poland. Results: A total of 586 individuals were included in the analysis. Individuals who were at a nutritional risk represented less than 2% of the study population, but more than 60% were overweight or obese. The mean BMI was 28.4 kg/m^2^ (SD: 5.16). LOS averaged 3.53 days (SD = 2.78). In the case of obese individuals, hospitalisation lasted for 3.4 ± 2.43 days, which was significantly longer than for patients of normal weight. For underweight patients, hospitalisation lasted for 5.14 ± 2.27 days, which was also significantly longer than for those in other BMI categories (*p* = 0.017). The independent predictors of shorter hospitalisations involved higher LDL concentration (parameter of regression: −0.015) and HDL concentration (parameter of regression: −0.04). Conclusions: The study revealed that with regard to the nutritional status of hypertensive patients, being either underweight or obese was associated with longer LOS. Additional factors that related to prolonged LOS were lower LDL and HDL levels and higher CRP concentrations.

## 1. Introduction

Cardiovascular diseases (CVDs) are the leading cause of death worldwide. According to the World Health Organization, nearly 18 million people died from CVDs in 2019, which accounted for 32% of all deaths worldwide [1]. Hypertension affects 40–45% of the adult population worldwide. It is a modifiable CVD risk factor and its increase exhibits a linear relationship with mortality and the development of other conditions, such as myocardial infarction, heart failure, and cerebral stroke. CVD treatment is costly and undoubtedly constitutes a global public health problem [2,3]. Some of the factors that affect the length of hospital stay (LOS) of a patient include both poor nutritional status and the presence of comorbidities [4,5]. A malnourished patient is at a higher risk of complications and the effectiveness of their treatment is lower. Additionally, a poor nutritional status results in prolonged LOS, thereby increasing the cost of treatment. It should be emphasised that it is not only being underweight that may cause the exacerbation of chronic diseases during hospital stays and worsen the prognoses of patients, but also being overweight [6]. Obesity is a particularly dangerous problem because its accompanying nutritional disorders are too rarely considered. Obese patients, especially those who have other chronic diseases, such as diabetes mellitus or chronic kidney disease, are wrongly assumed not to be at risk of having poor nutritional status. However, there is the possibility of quality malnutrition for overweight and obese patients who have additional hypertension. Ample scientific evidence has indicated that excessive body weight and visceral fat are the main causes of hypertension in up to 65–75% of cases [7]. Complications and comorbidities that are associated with hypertension, including obesity, increase the likelihood of hospitalisation [8]. Abnormal nutritional status is also associated with higher chances of complications, longer LOS, and higher mortality rates [9]. According to the Global Leadership Initiative on Malnutrition (GLIM) report and current Polish legislation, each patient who is admitted to hospital should be routinely assessed for nutritional status using the recommended tools, such as the Nutritional Risk Score 2002 (NRS 2002) [10,11]. Despite healthcare workers being key to the promotion of healthy lifestyles and the development of options for implementing nutritional interventions, the number of both malnourished patients and those at risk of malnutrition is increasing [12]. There has been a lot of evidence to suggests that the integration of healthcare that provides patient-centred care, such as the chronic care model (CCM), could be a solution in terms of reducing the rates of mortality and avoidable hospitalisations and improving clinical parameters [13,14]. Few studies have addressed the nutritional status of patients who have been diagnosed with hypertension and its impact on LOS.

This study aimed to assess how nutritional status and body mass index (BMI) affect LOS in patients who have hypertension.

## 2. Materials and Methods

### 2.1. Study Design and Setting

We performed a retrospective analysis of 586 medical records of patients who were admitted to the Institute of Heart Diseases of the University Clinical Hospital in Wroclaw, Poland, for hypertension (ICD10:I10) between January 2017 and June 2021.The study followed the guidelines of Strengthening the Reporting of Observational Studies in Epidemiology.

### 2.2. Study Population

We analysed all patients who met the inclusion criteria (diagnosis of hypertension and an age of ≥18 years). Finally, the medical records of 586 patients were examined. We investigated data such as sex, age, hypertension grade (according to the European Society of Cardiology/European Society of Hypertension guidelines), and BMI (kg/m^2^); comorbidities and medical history, including heart failure, diabetes mellitus, chronic kidney disease, cerebral stroke, and myocardial infarction; results of laboratory tests for triglycerides, low-density lipoprotein (LDL), high-density lipoprotein (HDL), total cholesterol, C-reactive protein (CRP), albumins, transferrin, lymphocytes, procalcitonin, potassium, sodium, haemoglobin A1c; and nutritional risk using the NRS 2002. The parameters were measured at the time of the admission to the cardiology department.

### 2.3. Nutritional Risk Score

The NRS 2002 is one of the screening tools that are recommended by GLIM [10]. It is based on impaired nutritional status (weight loss, BMI, and food intake during the preceding week), disease severity, and age. Patients are classified as either being at a nutritional risk (≥3 points) or not (<3 points) [11]. The criteria from the Word Health Organization were used to classify patients as underweight (BMI < 18.5), normal weight (BMI 18.5–24.9), pre-obese (BMI 25–29.9), and obese (BMI ≥ 30). A physician established the NRS 2002 status and BMI of the patients at admission to the cardiology department.

### 2.4. Statistical Analyses

The distributions of the quantitative variables were summarised with means, standard deviations, medians, and quartiles, whereas the distributions of the qualitative variables were summarised with the number and percentage of occurrence for each of their values. The chi-squared test (with Yates’ correction for 2 × 2 tables) was used to compare the qualitative variables of the groups. In the case of low values in the contingency tables, the Fisher’s exact test was applied instead. The Mann–Whitney test served to compare the quantitative variables of two groups, while the Kruskal–Wallis test (followed by Dunn’s post hoc test) was used for comparisons between more than two groups. The relationship between two quantitative variables was assessed using the Spearman’s correlation coefficient. Linear regressions were used to analyse the impact of potential predictors on the quantitative variables. Regression parameters with 95% confidence intervals were shown. Variables in multiple regression were selected on the basis of their significance in the simple regressions. Variables with the lowest *p* values were chosen so that the subjects per variable index equalled at least 10. The significance level for all statistical tests was set at 0.05. The R 4.1.2 (R Core Team (2021). R: A language and environment for statistical computing. R Foundation for Statistical Computing, Vienna, Austria URL https://www.R-project.org, accessed on 1 February 2022) software was used for the computations.

## 3. Results

### 3.1. Study Group Characteristics

The characteristics of the group are shown in Table 1 (qualitative variables) and Table 2 (quantitative variables). A total of 586 individuals were included in the analysis. Owing to missing data for some parameters, their counts were smaller, as provided with each variable. Women constituted 54.78% of the study group. The mean age equalled 63 years (SD: 12.7). Individuals who were at a nutritional risk represented less than 2% of the study population, but more than 60% were overweight or obese. The mean BMI was 28.4 kg/m^2^ (SD: 5.16).

### 3.2. Characteristics of the Study Group According to BMI

The characteristics of the study group according on BMI score are shown in Table 3. Both triglycerides and CRP were statistically significantly higher in obese patients than in the overweight and normal weight groups and were also significantly higher in the overweight group than in the normal weight group (*p* < 0.001). HDL concentration was significantly higher in the normal weight group than in overweight and obese patients and was also significantly higher in the overweight group than in the obese group (*p* < 0.001). Albumin concentration was significantly higher in obese and normal weight individuals than in the underweight group (*p* = 0.048). Haemoglobin A1c was significantly higher in the obese and underweight groups than in the normal weight group (*p* = 0.04). The risk of malnutrition, in accordance with the NRS scale, was significantly higher in underweight patients (*p* = 0.008). Diabetes mellitus and chronic kidney disease were also more frequent in this group.

### 3.3. Characteristics of the Study Group According to the NRS 2002

The characteristics of the study group according on the NRS 2002 scores are presented in Table 4. BMI, LDL, total cholesterol, albumin, and lymphocytes were significantly higher in the group of patients who were not at risk of malnutrition. However, age and procalcitonin levels were significantly higher in patients who were at a risk of malnutrition.

### 3.4. Length of Hospital Stay

LOS averaged 3.53 days (SD = 2.78). The shortest hospitalisation lasted for 1 day and the longest lasted for 21 days. LOS was significantly longer for patients with grade 3 hypertension (4.2 ± 2.6 days, *p* < 0.001) than for those with grade 1 or 2 hypertension. In the case of obese individuals, hospitalisation lasted for 3.4 ± 2.43 days, which was significantly longer than in the case of patients of normal weight. For underweight patients, hospitalisation lasted for 5.14 ± 2.27 days, which was also significantly longer than for patients in the other BMI categories (*p* = 0.017). LOS was significantly longer for the group with heart failure (4.43 ± 2.98 days, *p* < 0.001) vs. the group without, the group with chronic kidney disease (4.47 ± 3.68 days, *p* = 0.004) vs. the group without, the group with myocardial infarction (4.4 ± 2.87 days, *p* = 0.009) vs. the group without, patients with LDL < 70 mg/dL (4.45 ± 3.78 days, *p* < 0.001) vs. those with LDL ≥ 70 mg/dL, and individuals with HDL < 40 mg/dL (4.4 ± 3.22 days, *p* < 0.001) vs. those with HDL ≥ 40 mg/dL (Table 5).

In terms of numerical traits, hospitalisation correlated significantly and positively (r > 0) with age and CRP, i.e., the older the patient and the higher the CRP, the longer the hospitalisation. Hospitalisation correlated significantly and negatively (r < 0) with LDL, HDL, and albumin, i.e., the higher the values of those parameters, the shorter the hospitalisation (Table 6).

The multivariate linear regression model revealed that the independent predictors of longer hospitalisations included coexisting chronic kidney disease (parameter of regression = 0.914, *p* = 0.043) and a higher CRP concentration (parameter of regression = 0.013, *p* = 0.049). The independent predictors of shorter hospitalisations included a higher LDL concentration (parameter of regression = −0.015) and a higher HDL concentration (parameter of regression = −0.04). Being male sex shortened LOS by approximately 0.7 days compared to being female (Table 7).

## 4. Discussion

CVDs are a significant public health challenge and burden worldwide. The available global data indicate that poor nutritional status is common among patients in many hospital wards [15]. Nutritional status is often not considered during clinical practice and, as a result, a high proportion of patients who have CVDs also remain with undiagnosed malnutrition [16]. Nutritional status is one of the factors that can worsen prognoses and affect LOS and patient survival [17,18]. It is well known that BMI is strongly associated with hypertension. In this study, the mean BMI was over 28 kg/m^2^ and more than 75% of subjects were classified as overweight or obese. Landi et al. [19] reported a similar mean BMI in their study (26.7 kg/m^2^). Total cholesterol levels were also similar in both studies and equalled 192 and 211 mg/dL, respectively. High total cholesterol levels (>200 mg/dL) were noted by Gupta et al. [20] in 25% of patients with hypertension in their study. Research has confirmed that an increase in abdominal fat is positively associated with hypertension. Being overweight or obese can lead to hypertension and play a key role in its pathogenesis [21,22]. Numerous studies have implied that increased body fat is an independent risk factor for hypertension; however, the mechanisms of this relationship are not fully understood [21,23]. Inflammatory processes also play an important role in hypertension and it is known that fat cells produce a large number of proinflammatory cytokines. This inflammatory response is involved in the elevation of blood pressure [24,25].

In obese and overweight patients, significantly higher triglycerides and CRP concentrations were recorded compared to the normal weight group. On the other hand, the HDL fraction was higher in individuals with lower body weights. Many studies have confirmed the association between high BMI scores and abnormalities in these parameters and obesity, hypertriglyceridemia, and elevated CRP are also associated [26]. It is worth noting that increased CRP levels may indicate inflammation in the arterial walls of overweight and obese patients. When chronic, this situation may initiate vascular atherosclerosis, even in the absence of lipogram abnormalities [27]. Numerous studies have also demonstrated an association between increased LDL levels and hypertension [28,29]. Other investigators have confirmed that higher CRP levels are an independent risk factor for hypertension [30,31].

We found that albumin levels were statistically significantly lower in patients who were at a risk of malnutrition, in accordance with the NRS 2002, than in those with a BMI > 18.5 kg/m^2^ and averaged 2.43 g/dL. Albumin is a typical marker that is used to assess malnutrition, but its concentration may not only be affected by protein intake but also by overhydration, inflammation or other factors [32]. Studies have shown that a drop in serum albumin levels to below 3.5 g/dL increases the risk of death by a factor of four compared to individuals with levels above this value. A concentration of less than 3 g/dL is considered to be critical [33].

The performed univariate analysis revealed several factors that were statistically significantly associated with extended LOS. For parameters that were related to nutritional status, more problems occurred among patients with a BMI of <18.5 kg/m^2^ compared to those with higher values and among patients with obesity compared to those of normal weight. Allard et al. [34] analysed patient data from 18 Canadian hospitals and also observed that the risk of malnutrition on admission was independently associated with prolonged LOS. A BMI of <18.5 kg/m^2^ qualifies a patient for malnutrition status when they are also at risk for malnutrition in accordance with an assessment using a validated tool [35]. It is worth remembering that BMI is not an ideal index as it does not take into account individual components of body mass. BMI alone does not necessarily indicate malnutrition, e.g., for patients with coexisting heart failure, their BMI result may be higher on admission owing to fluid retention. However, it is a simple and widely available tool [36]. Epidemiological studies have shown that both low and high BMI levels are associated with increased morbidity and mortality from various causes [37]. Our results regarding BMI were similar to those obtained by Kyle et al. [38], who demonstrated that increased LOS was caused by obesity and low muscle mass, among other factors.

Our multivariate analysis indicated that the factors that affected LOS involved LDL levels of <70 mg/dL, HDL levels of <40 mg/dL, and increased CRP levels. Our multivariate model confirmed the association between these parameters and LOS. Research has demonstrated that for CVDs, an increase in CRP is associated with patient prognosis. In addition to being a biomarker for inflammation and a proatherosclerotic and prothrombotic factor, CRP may also constitute a predictor of other conditions, such as myocardial infarction, stroke, and sudden death [39]. When a seemingly healthy patient exhibits a high LDL level in addition to having hypertension and an increased CRP, there is an increased risk of myocardial infarction and stroke [40]. Ueda et al. [41] proved that moderately elevated blood pressure and LDL concentration over a long period has the same effect on the risk of ischemic heart disease as a short-term exposure to high LDL concentration and high blood pressure. Such studies have emphasised the importance of lifestyle modification as a primary prevention.

However, in our study, lower LDL and HDL levels were associated with prolonged hospitalisation. More than 75% of the patients in our study were overweight or obese. Some studies have shown that patients who struggle with obesity are up to 10 years younger than those with normal weight. This may be one of the reasons that physicians step up their treatment. These patients may also be at a high risk of CVDs and receive, for example, medication to lower their LDL levels [42]. In the case of low HDL levels, it is important to note that obesity is a significant contributing factor. Low HDL concentrations predispose an increased risk of CVDs. In a study by Bora et al. [43], a decrease of 79.8% in HDL level in overweight or obese subjects was associated with an increase in BMI–in overweight or obese subjects. Weight reduction may improve HDL levels and lower blood pressure.

Patients with obesity also present with more comorbidities [44]. Therefore, LOS and associated costs increase with the number of hospitalisations. Naturally, patients with multimorbidity and complications require longer care and additional resources [45]. Studies have shown that multimorbidity is an independent risk factor for complications, longer LOS, and higher mortality rates [46]. CVDs, including hypertension, are predictors of longer LOS among cardiac patients [47].

### Study Limitations

This study had some limitations. The first was the small group of patients who had an increased risk of malnutrition. They constituted less than 2% of the study group. The long-term survival of hypertensive patients could not be assessed because of data limitations that were due to the anonymity of the medical records. In some cases, the NRS and BMI scores were not reported in the medical records. The records also missed information on previous treatment, e.g., patients receiving lipid-lowering medication. Additionally, the individuals were not screened for body composition analysis and BMI is not a reliable measure of being overweight or obese. The patients also did not have their waist-to-hip circumference ratio examined, nor were other relevant data recorded, such as central obesity based on waist circumference.

## 5. Conclusions

The study revealed that with regard to the nutritional status of hypertensive patients, being either underweight (BMI < 18.5 kg/m^2^) or obese (BMI ≥ 30 kg/m^2^) was associated with longer LOS. Additional factors that were related to prolonged LOS were lower LDL and HDL levels and higher CRP concentrations. There is a need for further investigations into the nutritional status of hypertension patients who have been hospitalised in cardiac departments.

## Figures and Tables

**Table 1:** Study group characteristics (qualitative variables).

Parameter		Total (N = 586)
Sex	Female	321 (54.78%)
	Male	265 (45.22%)
Hypertension Grade	1	121 (20.65%)
	2	298 (50.85%)
	3	111 (18.94%)
	Unknown	56 (9.56%)
NRS 2002	<3	449 (76.62%)
	≥3	11 (1.88%)
	Unknown	126 (21.50%)
BMI (kg/m^2^)	<18.5	7 (1.19%)
	18.5–24.9	114 (19.45%)
	25.0–29.9	187 (31.91%)
	≥30	181 (30.89%)
	Unknown	97 (16.55%)
HF	No	502 (85.67%)
	Yes	84 (14.33%)
DM	No	431 (73.55%)
	Yes	155 (26.45%)
CKD	No	507 (86.52%)
	Yes	79 (13.48%)
CS	No	506 (86.35%)
	Yes	80 (13.65%)
MI	No	538 (91.81%)
	Yes	48 (8.19%)
TG (mg/dL)	<135 mg/dL	357 (60.92%)
	135–200 mg/dL	127 (21.67%)
	>200 mg/dL	73 (12.46%)
	Unknown	29 (4.95%)
LDL (mg/dL)	<70 mg/dL	65 (11.09%)
	70–116 mg/dL	181 (30.89%)
	>116 mg/dL	308 (52.56%)
	Unknown	32 (5.46%)
HDL (mg/dL)	<40 mg/dL	87 (14.85%)
	≥40 mg/dL	470 (80.20%)
	Unknown	29 (4.95%)

NRS 2002, Nutritional Risk Score 2002; BMI, body mass index; HF, heart failure; DM, diabetes mellitus; CKD, chronic kidney disease; CS, cerebral stroke; MI, myocardial infraction; TG, triglycerides; LDL, low-density lipoprotein; HDL, high-density lipoprotein.

**Table 2:** Study group characteristics (quantitative variables).

Parameter	N	Missing	Mean	SD	Median	Min	Max	Q1	Q3
Age (years)	586	0	63.3	12.7	65	20	96	56	72
BMI (kg/m^2^)	489	97	28.8	5.16	28.4	14.4	48.1	25.1	32.18
TG (mg/dL)	557	29	133.4	70.2	117	37	564	88	156
LDL (mg/dL)	554	32	130.7	54.4	127	23	415	89	166
HDL (mg/dL)	557	29	53.3	14.4	52	9	118	44	61
TC (mg/dL)	559	27	192.1	51	187	54	415	156	226
CRP (mg/L)	497	89	7.2	23.3	2.03	0.15	321.3	1.01	4.24
Albumin (g/dL)	30	556	3.46	0.68	3.5	1.8	4.5	2.92	3.88
Transferrin (g/L)	33	553	2.4	0.65	2.27	0.93	3.84	2.02	2.77
Lymphocytes (%)	112	474	25.8	9.09	25.7	3.4	56.1	19.9	31.5
PCT (ng/mL)	56	530	2.27	8.06	0.05	0.01	50	0.02	0.28
K (mmol/L)	580	6	4.25	0.51	4.22	2.82	7.37	3.96	4.48
Na (mmol/L)	580	6	139.9	3.04	140	110	152	139	142
HbA1c (%)	416	170	6.07	0.96	5.8	4.3	10.7	5.5	6.2

BMI, body mass index; TG, triglycerides; LDL, low-density lipoprotein; HDL, high-density lipoprotein; TC, total cholesterol; CRP, C-reactive protein; PCT, procalcitonin; K, potassium; Na, sodium; HbA1c, haemoglobin A1c.

**Table 3:** Comparison of the assessed parameters according to BMI status (qualitative and quantitative variables).

Parameter		BMI (kg/m^2^)				*p*-Value
		<18.5 (A) (N = 7)	18.5–24.9 (B) (N = 114)	25.0–29.9 (C) (N = 187)	≥30 (D) (N = 181)	
Age (years)	Mean ± SD	62.4 ± 14.2	62.9 ± 12.9	63.4 ± 13.72	62.1 ± 10.8	0.389
	Median	66	64	66	64	
	Quartiles	52–70.5	56.3–72	56–73	55–69	
TG (mg/dL)	Mean ± SD	100.7 ± 32.9	110.5 ± 56.2	128.8 ± 60.7	147.3 ± 74.8	<0.001 *
	Median	100	102.5	116	127	
	Quartiles	85.5–121	73.3–131	81.5–157.5	98.3–167.5	C > B; D > C, B
LDL (mg/dL)	Mean ± SD	89.7 ± 51.3	135.1 ± 55.1	133.3 ± 57.2	130.9 ± 54.6	0.19
	Median	70	141	130.5	123	
	Quartiles	61–123	87.5–174	90.3–164.8	89–164	
HDL (mg/dL)	Mean ± SD	47.4 ± 24.42	57.9 ± 13.6	54.5 ± 15.1	49.9 ± 12.1	<0.001 *
	Median	47	57	52	49.5	
	Quartiles	31.5–59.5	47–69.3	45–62	41–57	B > C, D; C > D
TC (mg/dL)	Mean ± SD	155 ± 72.1	194.9 ± 54.2	194.3 ± 51.3	188 ± 48.6	0.247
	Median	130	184	189	184	
	Quartiles	121.5–190	154.3–231.8	158.5–226	151–216	
CRP (mg/L)	Mean ± SD	46.9 ± 102.1	2.34 ± 3.14	10.3 ± 33.8	6.37 ± 11.21	<0.001 *
	Median	5.11	1.4	1.97	2.71	
	Quartiles	1.46–12.9	0.6–2.35	1.11–3.83	1.31–5.85	C > B; D > C, B
Albumin (g/dL)	Mean ± SD	2.43 ± 0.57	3.77 ± 0.59	3.52 ± 0.54	3.79 ± 0.57	0.048 *
	Median	2.6	3.8	3.45	3.7	
	Quartiles	2.2–2.75	3.7–4.12	3.2–3.65	3.55–4.15	B, D > A
Transferrin (g/L)	Mean ± SD	0.93 ± NA	2.47 ± 0.58	2.43 ± 0.55	2.52 ± 0.71	0.42
	Median	0.93	2.3	2.27	2.31	
	Quartiles	0.93–0.93	2.13–2.77	2.08–2.55	1.89–3.08	
Lymphocytes (%)	Mean ± SD	11.9 ± 6.12	27.3 ± 9.52	24.5 ± 8.91	25.3 ± 6.47	0.046 *
	Median	11	24.65	25.45	25.7	
	Quartiles	8.22–14.7	22.2–36.8	19.9–31.4	20.9–28.7	D, C, B > A
PCT (ng/mL)	Mean ± SD	23.1 ± 24.81	0.14 ± 0.21	0.54 ± 1.2	0.47 ± 1.32	0.04 *
	Median	18.15	0.03	0.07	0.04	
	Quartiles	9.64–34.1	0.01–0.16	0.03–0.18	0.03–0.12	A > C, D, B
K (mmol/L)	Mean ± SD	4.25 ± 0.51	4.29 ± 0.51	4.29 ± 0.57	4.24 ± 0.45	0.883
	Median	4.51	4.24	4.22	4.22	
	Quartiles	3.9–4.6	3.96–4.52	3.96–4.48	3.98–4.43	
Na (mmol/L)	Mean ± SD	134.6 ± 5.26	140.2 ± 2.68	139.9 ± 3.03	140.1 ± 2.38	0.057
	Median	133	140	140	140	
	Quartiles	131.5–138	139–142	139–142	139–142	
HbA1c (%)	Mean ± SD	7.47 ± 2.05	5.95 ± 0.91	5.97 ± 0.81	6.21 ± 1.1	0.04 *
	Median	6.9	5.7	5.8	5.9	
	Quartiles	5.85–9.3	5.5–6.18	5.5–6.1	5.5–6.3	A, D > B
Sex	Female	4 (57.14%)	69 (60.53%)	92 (49.20%)	94 (51.93%)	0.274
	Male	3 (42.86%)	45 (39.47%)	95 (50.80%)	87 (48.07%)	
Hypertension Grade	1	2 (40.00%)	28 (26.92%)	36 (22.22%)	32 (19.05%)	0.687
	2	2 (40.00%)	59 (56.73%)	95 (58.64%)	99 (58.93%)	
	3	1 (20.00%)	17 (16.35%)	31 (19.14%)	37 (22.02%)	
NRS	<3	5 (71.43%)	85 (96.59%)	146 (98.65%)	142 (98.61%)	0.008 *
	≥3	2 (28.57%)	3 (3.41%)	2 (1.35%)	2 (1.39%)	
HF	No	4 (57.14%)	102 (89.47%)	162 (86.63%)	153 (84.53%)	0.11
	Yes	3 (42.86%)	12 (10.53%)	25 (13.37%)	28 (15.47%)	
DM	No	4 (57.14%)	93 (81.58%)	136 (72.73%)	118 (65.19%)	0.013 *
	Yes	3 (42.86%)	21 (18.42%)	51 (27.27%)	63 (34.81%)	
CKD	No	4 (57.14%)	97 (85.09%)	156 (83.42%)	165 (91.16%)	0.018 *
	Yes	3 (42.86%)	17 (14.91%)	31 (16.58%)	16 (8.84%)	
CS	No	5 (71.43%)	97 (85.09%)	161 (86.10%)	161 (88.95%)	0.359
	Yes	2 (28.57%)	17 (14.91%)	26 (13.90%)	20 (11.05%)	
MI	No	6 (85.71%)	106 (92.98%)	175 (93.58%)	164 (90.61%)	0.477
	Yes	1 (14.29%)	8 (7.02%)	12 (6.42%)	17 (9.39%)	
TG	<135 mg/dL	6 (85.71%)	84 (77.78%)	115 (62.84%)	98 (57.65%)	0.005 *
	135–200 mg/dL	1 (14.29%)	18 (16.67%)	47 (25.68%)	39 (22.94%)	
	>200 mg/dL	0 (0.00%)	6 (5.56%)	21 (11.48%)	33 (19.41%)	
LDL	<70 mg/dL	3 (42.86%)	14 (13.08%)	22 (12.09%)	16 (9.47%)	0.055
	70–116 mg/dL	2 (28.57%)	26 (24.30%)	55 (30.22%)	63 (37.28%)	
	>116 mg/dL	2 (28.57%)	67 (62.62%)	105 (57.69%)	90 (53.25%)	
HDL	<40 mg/dL	3 (42.86%)	8 (7.41%)	22 (12.09%)	37 (21.76%)	0.001 *
	≥40 mg/dL	4 (57.14%)	100 (92.59%)	160 (87.91%)	133 (78.24%)	

BMI, body mass index; TG, triglycerides; LDL, low-density lipoprotein; HDL, high-density lipoprotein; TC, total cholesterol; CRP, C-reactive protein; NA, not available; PCT, procalcitonin; K, potassium; Na, sodium; HbA1c, haemoglobin A1c; NRS 2002, Nutritional Risk Score 2002; HF, heart failure; DM, diabetes mellitus; CKD, chronic kidney disease; CS, cerebral stroke; MI, myocardial infarction; *p*, Kruskal–Wallis test + post hoc analysis (Dunn’s test) for quantitative variables and chi-squared or Fisher’s exact test for qualitative variables; * statistically significant (*p* < 0.05).

**Table 4:** Comparison of the assessed parameters according to NRS 2002 status.

Parameter		NRS 2002		*p*-Value
		<3 (N = 449)	≥3 (N = 11)	
Age (years)	Mean ± SD	63.5 ± 12.4	74.5 ± 12	0.004 *
	Median	65	79	
	Quartiles	56–72	68–82.5	
BMI (kg/m^2^)	Mean ± SD	28.8 ± 5	25.2 ± 5.93	0.04 *
	Median	28.42	24.8	
	Quartiles	25.3–32.2	23.1–25.8	
TG (mg/dL)	Mean ± SD	135 ± 70	116 ± 29	0.695
	Median	119.5	126	
	Quartiles	90.3–160.3	86–135	
LDL (mg/dL)	Mean ± SD	126.5 ± 52.5	77.9 ± 36.7	0.003 *
	Median	120	79	
	Quartiles	86–160.3	57–89	
HDL (mg/dL)	Mean ± SD	53.2 ± 14.2	45.1 ± 20.2	0.244
	Median	52	47	
	Quartiles	43–61	27–61	
TC (mg/dL)	Mean ± SD	194.4 ± 50.2	146.2 ± 54.9	0.01 *
	Median	190	145	
	Quartiles	158.8–230.3	122–162	
CRP (mg/L)	Mean ± SD	7.3 ± 22.3	30.5 ± 75.5	0.294
	Median	2.04	4.31	
	Quartiles	1.07–4.94	1.22–9.21	
Albumin (g/dL)	Mean ± SD	3.62 ± 0.57	2.62 ± 0.59	0.005 *
	Median	3.7	2.6	
	Quartiles	3.2–4.1	2.4–2.9	
Transferrin (g/L)	Mean ± SD	2.45 ± 0.62	1.91 ± 1.05	0.334
	Median	2.32	1.77	
	Quartiles	2.07–2.73	1.35–2.4	
Lymphocytes (%)	Mean ± SD	25.8 ± 8.91	13 ± 10.3	0.028 *
	Median	25.55	9.1	
	Quartiles	19.9–31	8.22–13.9	
PCT (ng/mL)	Mean ± SD	1.16 ± 4.53	14.2 ± 21.4	0.006 *
	Median	0.04	1.42	
	Quartiles	0.02–0.17	1.21–18.2	
K (mmol/L)	Mean ± SD	4.26 ± 0.53	4.21 ± 0.77	0.907
	Median	4.23	4.28	
	Quartiles	3.96–4.5	3.8–4.5	
Na (mmol/L)	Mean ± SD	139.9 ± 3.11	138.18 ± 5.42	0.623
	Median	140	141	
	Quartiles	139–142	135.5–141.5	
HbA1c (%)	Mean ± SD	6.07 ± 0.95	6.53 ± 1.15	0.09
	Median	5.9	6.25	
	Quartiles	5.5–6.2	6.12–6.3	
Sex	Female	247 (55.01%)	7 (63.64%)	0.761
	Male	202 (44.99%)	4 (36.36%)	
Hypertension Grade	1	91 (22.69%)	2 (22.22%)	0.315
	2	221 (55.11%)	3 (33.33%)	
	3	89 (22.19%)	4 (44.44%)	
BMI (kg/m^2^)	Underweight	5 (1.32%)	2 (22.22%)	0.008 *
	Normal	85 (22.49%)	3 (33.33%)	
	Overweight	146 (38.62%)	2 (22.22%)	
	Obese	142 (37.57%)	2 (22.22%)	
HF	No	381 (84.86%)	10 (90.91%)	1
	Yes	68 (15.14%)	1 (9.09%)	
DM	No	326 (72.61%)	7 (63.64%)	0.505
	Yes	123 (27.39%)	4 (36.36%)	
CKD	No	384 (85.52%)	6 (54.55%)	0.016 *
	Yes	65 (14.48%)	5 (45.45%)	
CS	No	396 (88.20%)	9 (81.82%)	0.629
	Yes	53 (11.80%)	2 (18.18%)	
MI	No	411 (91.54%)	9 (81.82%)	0.247
	Yes	38 (8.46%)	2 (18.18%)	
TG	<135 mg/dL	263 (61.74%)	6 (66.67%)	0.625
	135–200 mg/dL	107 (25.12%)	3 (33.33%)	
	>200 mg/dL	56 (13.15%)	0 (0.00%)	
LDL	<70 mg/dL	52 (12.26%)	3 (33.33%)	0.016 *
	70–116 mg/dL	152 (35.85%)	5 (55.56%)	
	>116 mg/dL	220 (51.89%)	1 (11.11%)	
HDL	<40 mg/dL	71 (16.67%)	4 (44.44%)	0.052
	≥40 mg/dL	355 (83.33%)	5 (55.56%)	

NRS 2002, Nutritional Risk Score 2002; BMI, body mass index; TG, triglycerides; LDL, low-density lipoprotein; HDL, high-density lipoprotein; TC, total cholesterol; CRP, C-reactive protein; PCT, procalcitonin; K, potassium; Na, sodium; HbA1c, haemoglobin A1c; HF, heart failure; DM, diabetes mellitus; CKD, chronic kidney disease; CS, cerebral stroke; MI, myocardial infarction; *p*, Mann–Whitney test for quantitative variables and chi-squared or Fisher’s exact test for qualitative variables; * statistically significant (*p* < 0.05).

**Table 5:** Length of hospital stay across groups (qualitative variables): univariate analysis.

Parameter	Group	Hospitalisation (Days)			*p*-Value
		Mean ± SD	Median	Quartiles	
Sex	Female (N = 321)	3.65 ± 2.83	3	1–5	0.261
	Male (N = 265)	3.39 ± 2.72	3	1–4	
Hypertension Grade	1 (N = 121) (A)	3.6 ± 3.13	3	1–5	<0.001 *
	2 (N = 298) (B)	3.2 ± 2.58	3	1–4	
	3 (N = 111) (C)	4.2 ± 2.6	4	3–6	C > A, B
NRS	<3 (N = 449)	3.73 ± 2.58	3	2–5	0.078
	≥3 (N = 11)	6.82 ± 6.16	5	2.5–8	
BMI	<18.5 (N = 7) (A)	5.14 ± 2.27	6	4.5–6.5	0.017 *
	18.5–24.9 (N = 114) (B)	2.98 ± 2.71	2	1–4	
	25.0–29.9 (N = 187) (C)	3.3 ± 2.83	3	1–4	D > B
	≥30 (N = 181) (D)	3.4 ± 2.43	3	1–5	A > D, C, B
HF	No (N = 502)	3.38 ± 2.72	3	1–5	<0.001 *
	Yes (N = 84)	4.43 ± 2.98	4	3–5.25	
DM	No (N = 431)	3.45 ± 2.71	3	1–5	0.233
	Yes (N = 155)	3.75 ± 2.98	3	1–5	
CKD	No (N = 507)	3.38 ± 2.59	3	1–5	0.004 *
	Yes (N = 79)	4.47 ± 3.68	3	2.5–5.5	
CS	No (N = 506)	3.49 ± 2.74	3	1–5	0.67
	Yes (N = 80)	3.78 ± 3.02	3	1–5	
MI	No (N = 538)	3.45 ± 2.76	3	1–5	0.009 *
	Yes (N = 48)	4.4 ± 2.87	4	2.75–6	
TG	<135 mg/dL (N = 357)	3.47 ± 2.82	3	1–5	0.594
	135–200 mg/dL (N = 127)	3.54 ± 2.72	3	1.5–5	
	>200 mg/dL (N = 73)	3.62 ± 2.76	3	2–4	
LDL	<70 mg/dL (N = 65) (A)	4.45 ± 3.78	3	2–5	<0.001 *
	70–116 mg/dL (N = 181) (B)	4.13 ± 2.5	4	3–5	
	>116 mg/dL (N = 308) (C)	2.86 ± 2.36	2	1–4	B, A > C
HDL	<40 mg/dL (N = 87)	4.4 ± 3.22	4	3–6	<0.001 *
	≥40 mg/dL (N = 470)	3.29 ± 2.54	3	1–5	

NRS 2002, Nutritional Risk Score 2002; BMI, body mass index; HF, heart failure; DM, diabetes mellitus; CKD, chronic kidney disease; CS, cerebral stroke; MI, myocardial infarction; TG, triglycerides; LDL, low-density lipoprotein; HDL, high-density lipoprotein; *p*, Mann–Whitney test for comparisons of two groups and Kruskal–Wallis test plus post hoc analysis (Dunn’s test) for comparisons of more than two groups; * statistically significant (*p* < 0.05).

**Table 6:** Length of hospital stay (quantitative variables): univariate analysis.

Parameter	Hospitalisation
	Spearman’s Correlation Coefficient
Age (years)	r = 0.116, *p* = 0.005 *
BMI (kg/m^2^)	r = 0.039, *p* = 0.387
TG (mg/dL)	r = 0.06, *p* = 0.155
LDL (mg/dL)	r = −0.362, *p* < 0.001 *
HDL (mg/dL)	r = −0.178, *p* < 0.001 *
TC (mg/dL)	r = −0.067, *p* = 0.113
CRP (mg/L)	r = 0.202, *p* < 0.001 *
Albumin (g/dL)	r = −0.46, *p* = 0.01 *
Transferrin (g/L)	r = −0.305, *p* = 0.084
Lymphocytes (%)	r = −0.152, *p* = 0.11
PCT (ng/mL)	r = 0.235, *p* = 0.082
K (mmol/L)	r = −0.018, *p* = 0.667
Na (mmol/L)	r = −0.029, *p* = 0.479
HbA1c (%)	r = −0.008, *p* = 0.879

BMI, body mass index; TG, triglycerides; LDL, low-density lipoprotein; HDL, high-density lipoprotein; TC, total cholesterol; CRP, C-reactive protein; PCT, procalcitonin; K, potassium; Na, sodium; HbA1c, haemoglobin A1c; * statistically significant (*p* < 0.05).

**Table 7:** Multivariate linear regression model.

Trait		Parameter	95% CI		*p*-Value
Hypertension Grade	1	Ref.			
	2	−0.579	−1.418	0.261	0.178
	3	0.122	−0.881	1.126	0.811
CKD	No	Ref.			
	Yes	0.914	0.034	1.795	0.043 *
MI	No	Ref.			
	Yes	0.799	−0.383	1.982	0.186
Age	(years)	0.005	−0.023	0.032	0.731
LDL	(mg/dL)	−0.015	−0.024	−0.007	0.001 *
HDL	(mg/dL)	−0.04	−0.067	−0.013	0.004 *
CRP	(mg/L)	0.013	0	0.025	0.049 *
NRS	Not at nutritional risk	Ref.			
	At nutritional risk	1.695	−0.359	3.748	0.107
BMI	Normal	Ref.			
	Underweight	0.869	−1.881	3.619	0.536
	Overweight	0.025	−0.825	0.875	0.955
	Obese	−0.064	−0.918	0.79	0.883
TC	(mg/dL)	0.008	−0.002	0.018	0.115
DM	No	Ref.			
	Yes	0.338	−0.362	1.038	0.345
Sex	Female	Ref.			
	Male	−0.701	−1.358	−0.045	0.037 *

CKD, chronic kidney disease; MI, myocardial infarction; LDL, low-density lipoprotein; HDL, high-density lipoprotein; CRP, C-reactive protein; NRS 2002, Nutritional Risk Score 2002; BMI, body mass index; TC, total cholesterol; DM, diabetes mellitus; * statistically significant (*p* < 0.05).

## Data Availability

The data can be accessed by contacting the corresponding author.
